# Masse pelvienne chez une jeune fille: penser à l'hématocolpos

**DOI:** 10.11604/pamj.2014.17.84.3760

**Published:** 2014-02-03

**Authors:** Amina Ben Salem, Sana Yahyaoui, Amal Messoud, Houda El Mhabrech, Rajaa Faleh, Chiraz Hafsa

**Affiliations:** 1 Service radiologie B, Centre de maternité et de néonatologie, Monastir, Tunisie; 2 Service de gynécologie-obstétrique, Centre de maternité et de néonatologie, Monastir, Tunisie

**Keywords:** Hématocolpos, aménorrhée, échographie, imagerie par résonance magnétique, hematocolpos, amenorrhea, echography, MRI

## Abstract

L'hématocolpos est l'accumulation progressive du sang menstruel dans la cavité vaginale à la puberté. Il est souvent la conséquence d'une imperforation de l'hymen. Il se traduit sur le plan clinique par des douleurs pelviennes cycliques et une aménorrhée primaire. Plus rarement, il peut se révéler par une masse pelvienne. L’échographie est l'examen de choix pour le diagnostic de l'hématocolpos sur imperforation de l'hymen. L'imagerie par résonance magnétique (IRM) est l'examen d'imagerie de référence pour confirmer l'hématolcolpos et exclure d'autres malformations du canal de Muller ou des malformations urologiques associées. Nous rapportons un cas d'hématocolpos secondaire à une imperforation hyménéale diagnostiqué chez une jeune fille présentant une aménorrhée primaire et une masse pelvienne. Le diagnostic était posé par l’échographie et l'IRM et confirmé par l'intervention chirurgicale.

## Introduction

L'hématocolpos est une accumulation du sang menstruel dans la cavité vaginale. Il est en général du à une imperforation de l'hymen. L'imperforation de l'hymen est rarement diagnostiquée pendant la période néonatale et se présente en général plus tard à la puberté par une douleur pelvienne cyclique, une aménorrhée primaire ou une masse pelvienne. L'examen gynécologique permet d’évoquer le diagnostic de l'imperforation de l'hymen. L’échographie est déterminante pour le diagnostic d'hématocolpos. L'IRM permet de confirmer le diagnostic et de faire le bilan d'extension radiologique à la recherche d’éventuelles malformations uro-génitales associées.

## Patient et observation

**Figure 1:**
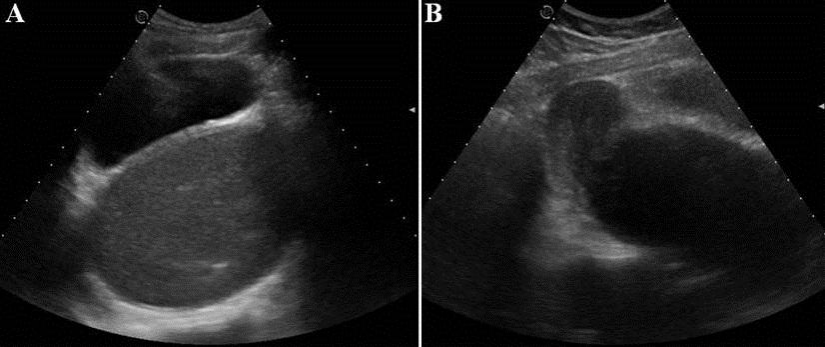
Echographie sus-pubienne: A: Coupe axiale: présence d'une formation liquidienne rétrovésicale, médiane, hypoéchogène renfermant un fin piqueté échogène; B: Coupe sagittale: la collection communique en haut avec la cavité utérine qui n'est pas distendue

**Figure 2:**
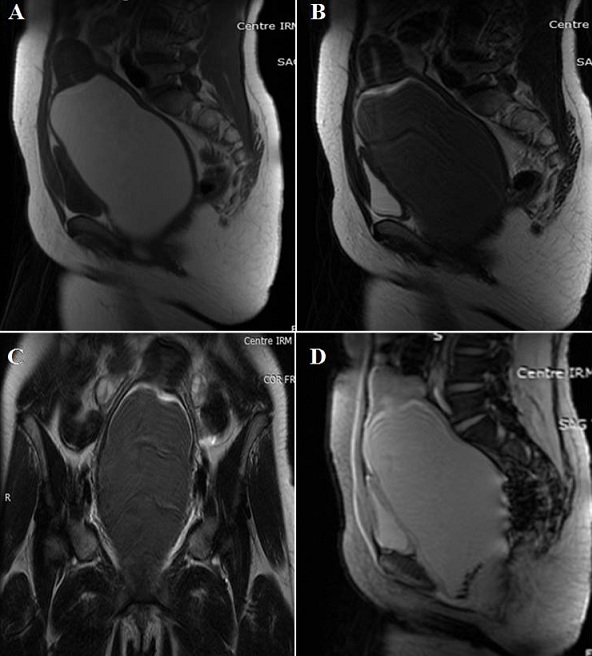
IRM pelvienne: A: séquence sagittale SE T1; B: séquence sagittale SE T2: importante distension du vagin à contenu en hypersignal T1, en hyposignal T2; C: séquence coronale SET2: la collection se continue en haut par l'utérus. Les ovaires sont de taille et de morphologie normales; D: séquence sagittale écho de gradient T2 (T2*): Présence de dépôts déclives en hyposignal T2* en rapport avec de l’'hémosidérine

Une fille âgée de 15 ans, n'ayant pas encore sa ménarche, était adressée à la consultation de gynécologie pour l'apparition progressive d'une masse pelvienne et de douleurs abdominales évoluant depuis 8 mois. A l'examen clinque, elle était apyrétique, normo-tendue. L'examen de l'abdomen montrait la présence d'une voussure pelvienne avec palpation d'une masse pelvienne médiane arrivant jusqu’à l'ombilic, sensible, ferme et mobile. Une origine tumorale était suspectée. L’échographie sus-pubienne a montré la présence d'une formation liquidienne rétrovésicale, médiane, hypoéchogène renfermant un fin piqueté échogène (Figure 1A). Cette collection était surmontée par la cavité utérine communicante, qui est non distendue (Figure 1B). L'IRM a mis en évidence une importante distension du vagin à contenu hématique en hypersignal T1 (Figure 2A), en hyposignal T2 (Figure 2B, Figure 2C), avec présence de dépôts déclives en hyposignal T2* en rapport avec de l'hémosidérine (Figure 2D). L'utérus était refoulé vers le haut. La cavité utérine n’était pas distendue. Aucune autre anomalie utéro-annexielle n’était trouvée. Le diagnostic d'hématocolpos était évoqué. La patiente a été réexaminée et l'examen des organes génitaux externes avait objectivé un hymen imperforé et bombé. Le diagnostic retenu était un hématocolpos sur hymen imperforé. Une hyménéotomie a été faite sous anesthésie générale et 500 millilitres de sang « couleur chocolat » ont été vidés. Les suites opératoires étaient favorables.

## Discussion

L′hématocolpos est la rétention vaginale de la menstruation. Il se constitue à la puberté dès la première menstruation et l′imperforation hyménéale est l′étiologie la plus fréquente. Un diaphragme vaginal complet plus haut situé, ou une atrésie vaginale partielle sont des causes plus rares [1]. L'imperforation hyménale a une fréquence de 0.1% avec présence de cas familiaux [2]. La puberté reste la période la plus fréquente de la découverte d′un hématocolpos. Le développement normal des caractères sexuels secondaires coexiste avec une aménorrhée primaire. Les signes cliniques sont dominés par la présence de douleurs à caractère cyclique et qui peut, en cas d'hématométrie, s'accompagner de masse hypogastrique. Les douleurs sont pelviennes ou parfois pseudo-appendiculaires [2]. Les douleurs lombaires, des sciatalgies avec déficit sensitif et moteur du membre inférieur ont été rencontrées [1]. Les signes urinaires dus à une compression peuvent être à l′origine d′une rétention aiguë [3], d′une dysurie, d′une hydronéphrose bilatérale. Le diagnostic est facile à établir cliniquement. L'examen de la vulve montre l'obstruction de l'orifice vaginal par une membrane (hymen) mince, bombée chez une patiente qui n'a pas encore ses menstruations malgré le développement des caractères sexuels secondaires [1, 2]. Les examens d'imagerie sont importants pour redresser le diagnostic en cas de doute diagnostique. L’échographie sus-pubienne montre une image rétrovésicale finement échogène. Cette collection est surmontée par la cavité utérine communicante, qui est souvent de petite taille, dilatée par un contenu liquidien en cas d'hématomètrie [4]. Un hématosalpinx ou un épanchement péritonéal peuvent être trouvés [2]. L'examen échographique analyse aussi les malformations utérines. L′agénésie rénale est systématiquement recherchée surtout en cas de duplication génitale [1]. L’échographie peut montrer aussi une dilatation urétéro-pyélocalicielle en cas de compression des vois urinaires par l'hématoscopes [3]. L'IRM a comme l′échographie l′avantage de l′innocuité chez une jeune fille. Elle serait la meilleure technique d'exploration complémentaire donnant sur les séquences pondérées en T2 une très bonne analyse anatomique morphologique de la malformation. Les séquences pondérées en T1 confirmeront le contenu hématique dans le vagin et dans la corne utérine sus-jacente [4, 5]. Le traitement de l'hématocolpos dépend de son étiologie. En cas d'hymen imperforé, il consiste en une hyménéotomie ou une hyménectomie dont le but est de drainer l'hématocolpos [1, 2].

## Conclusion

L'hématocolpos est une affection rare qui doit être évoquée devant tout syndrome douloureux aigu abdominopelvien ou masse pelvienne de la fille en période pubertaire non encore réglée, d'autant plus que les douleurs sont cycliques et que le développement des caractères sexuels secondaires est normal. L'imagerie est indispensable en cas de doute diagnostique. Elle permet d'orienter le diagnostic, de rechercher les éventuelles malformations uro-génitales associées et les signes de compression notamment urinaires. Une prévention par un examen soigneux des organes génitaux des nouveau-nées est possible.
